# Supplementary material for: Content-rich biological network constructed by mining PubMed abstracts
Source: BMC Bioinformatics. 2004 Oct 8;5:147. doi: 10.1186/1471-2105-5-147 (PMC528731; doi:10.1186/1471-2105-5-147)
Supplement: Additional File 5 — The original Chilibot query results of the term "long-term potentiation (LTP)" and 22 other terms, limiting the latest references analyzed to the years 1990, 1995, 2000, and 2004. [file 1471-2105-5-147-S5.bz2 › chilibotAdditionalFile5/ltp2004/html/SYNAPSIN I_SYNAPTOPHYSIN.html]

 


 **SYNAPSIN I** and **SYNAPTOPHYSIN** 
  
Found 71 abstracts in PubMed,  **30 abstracts were retrieved and analyzed**.  


---

 Search Google  |
 PDF files only 
|  EDU domain only 

---

**Interactive relationship** (e.g. stimulation, inhibition, etc)

- After training, pp60c src from hippocampus showed enhanced interactions with synaptic proteins such as  **synapsin I** ,  **synaptophysin** , and the type 2 N methyl d aspartate receptor, as well as the cytoskeletal protein actin.  Ref: 10884433 Proc Natl Acad Sci U S A, 2000

**Parallel relationship** (e.g. studied together, co-existance, homology, etc.)

- Horseradish peroxidase filled vacuoles as wells as vesicles contained the synaptic vesicle membrane proteins VAMP II, synaptotagmin, SV2, and  **synaptophysin** , the vesicle associated proteins rab 3A and  **synapsin I** , and in addition SNAP 25.  Ref: 10579591 Neuroscience, 1999
- Decreased immunoreactivity for  **synaptophysin** ,  **synapsin I** , SNAP 25, and syntaxin I was observed in the cerebral cortex in every case, thus showing generalized reduction of proteins involved in exocytosis of synaptic vesicles in the brains of patients with spongiform encephalopathy.  Ref: 10343324 Neurobiol Dis, 1999
- Decreased FM 1 43 staining correlated with low  **synapsin I**  and  **synaptophysin**  immunoreactivities, suggesting that decreased presynaptic function was commensurate with synaptic disassembly.  Ref: 11826107 J Neurosci, 2002
- The formation of functional NMDA receptors also coincided with the appearance of  **synapsin I**  and  **synaptophysin** .  Ref: 11920728 J Neurobiol, 2002
- In order to characterize synaptic involvement in human spongiform encephalopathies, the expression of synaptic vesicle associated proteins,  **synaptophysin**  and  **synapsin I** , and presynaptic plasma membrane proteins, synaptosomal associated protein of 25 kDa SNAP 25 and syntaxin I, was examined in the brains of four patients who had suffered from sporadic Creutzfeldt Jakob disease.  Ref: 10343324 Neurobiol Dis, 1999
- Similar expression profiles for the two proteins were also observed in immunocytochemical studies of Xenopus spinal neurons in culture intense staining of  **synaptophysin**  was found on the first day, while  **synapsin I**  was not detected until after three days in culture.  Ref: 8895877 Neuroscience, 1996
- In fimbria and corpus callosum levels of synaptic proteins,  **synapsin I**  and  **synaptophysin**  were 15 20% of those in cortex.  Ref: 12969269 J Neurochem, 2003
- In the outer plexiform layer OPL,  **synaptophysin**  appeared at P4, while  **synapsin I**  and syntaxin appeared at P8.  Ref: 9352109 Brain Res Dev Brain Res, 1997
- Furthermore, expression of  **synaptophysin** ,  **synapsin I** , syntaxin I and SNAP 25 is the same in the cerebellum of irradiated and normal rats from this time to adulthood 3 months.  Ref: 10479063 Int J Dev Neurosci, 1999
- In addition,  **synaptophysin** ,  **synapsin I** , and PSD 95 immunoreactivities were significantly reduced in both the gray and white matter of the LSSC during acute EAE episodes and EAE relapses, but showed partial recovery during EAE remission and after EAE recovery.  Ref: 12707048 Am J Pathol, 2003
- The expression and localization of vesicle associated membrane protein VAMP, synaptic vesicle protein 2 SV2,  **synaptophysin** ,  **synapsin I** , and synaptosomal associated protein of 25 kDa SNAP 25 was assessed immunocytochemically.  Ref: 11807837 J Comp Neurol, 2002
- VAChT containing varicosities in the Renshaw cell area, with a probable origin from motoneuron axon collaterals, exhibited CSP,  **synapsin I**  II, and synaptobrevin 1 LI... syntaxin, synuclein,  **synapsin I** , synaptotagmin I, synaptotagmin I II,  **synaptophysin**  and synaptobrevin 2 LI.  Ref: 10421869 J Comp Neurol, 1999
- The amount of immunoreactive  **synapsin I** ,  **synaptophysin** , and synaptotagmin was not appreciably changed for 72 hours after reperfusion.  Ref: 11988617 Stroke, 2002
- The presence of synaptic vesicle associated proteins such as  **synaptophysin** , synaptotagmin and  **synapsin I**  in the growth cones of regenerating axons indicates the possibility that exocytotic fusion of vesicles with the surface axolemma supplies the membranous components for the extension of regenerating axons.  Ref: 8829147 Neurosci Res, 1996
- The distribution of four proteins associated with synaptic vesicles, SV2,  **synaptophysin** ,  **synapsin I** , and rab3a, was investigated during postnatal development of the posteromedial barrel subfield in the rat somatosensory cortex.  Ref: 8915833 J Comp Neurol, 1996
- Photoreceptor terminals in the intact retina contain VAMP, SV2,  **synaptophysin** , and SNAP 25, but not  **synapsin I** .  Ref: 11807837 J Comp Neurol, 2002
- Immunohistochemistry in combination with confocal laser microscopy showed the presence of synaptosomal associated protein of 25 kDa, syntaxin, cysteine string protein, synuclein,  **synapsin I** ,  **synapsin I**  II, synaptotagmin I, synaptotagmin I II,  **synaptophysin** , and synaptobrevin 2 like immunoreactivity in VAChT containing C terminals.  Ref: 10421869 J Comp Neurol, 1999
- Dystrophic axons in a 2 year old male Rottweiler with neuroaxonal dystrophy have shown  **synaptophysin** ,  **synapsin I** , synaptosomal associated protein of 25 kDa SNAP 25, Rab 3a, and alpha synuclein immunoreactivity.  Ref: 11699565 Acta Neuropathol (Berl), 2001
- Expression of  **synaptophysin** ,  **synapsin I**  and syntaxin was studied immunocytochemically in the developing rat retina using indirect immunoperoxidase technique.  Ref: 9352109 Brain Res Dev Brain Res, 1997
- In parallel, there was a substantial increase in the expression levels of the presynaptic markers  **synaptophysin** , synaptotagmin, and  **synapsin I**  and of GluR1 AMPA receptor subunits.  Ref: 12595243 Mol Cell Neurosci, 2003
- Here, we show that inhibition of histone deacetylase activity in P19 cells is sufficient to activate transcription of the  **synaptophysin**  and  **synapsin I**  genes, indicating that neuronal differentiation and impairment of histone deacetylases results in a similar gene expression pattern.  Ref: 12492469 Eur J Biochem, 2003
- Determination of  **synapsin I**  and  **synaptophysin**  in body fluids by two site enzyme linked immunosorbent assays.  Ref: 9692851 J Immunol Methods, 1998
- Two site enzyme linked immunosorbent assays ELISA have been established for the specific and sensitive determination of two membrane proteins of the small synaptic vesicles SSV, namely peripheral  **synapsin I**  and integral  **synaptophysin** .  Ref: 9692851 J Immunol Methods, 1998
- In the inner plexiform layer IPL, syntaxin immunoreactivity appeared at postnatal day 1 P1 whereas  **synaptophysin**  and  **synapsin I**  staining were first observed at P2.  Ref: 9352109 Brain Res Dev Brain Res, 1997
- Specifically, syntaxin 1,  **synaptophysin**  and  **synapsin I**  protein levels were examined in the nucleus accumbens Nacc and ventral tegmental area VTA of Sprague Dawley rats following AMPH sensitization.  Ref: 11368965 Brain Res, 2001
- In the present study, immunohistochemistry to the presynaptic membrane proteins syntaxin I and synaptosomal associated protein of 25 kDa SNAP 25 synaptic vesicle associated proteins  **synaptophysin**  and  **synapsin I**  and the neuronal maturation and axonal growth related protein GAP 43, has been carried out in the normal developing cerebellum and following a single dose of ionizing radiation 2 Gy gamma rays at postnatal day 1.  Ref: 10479063 Int J Dev Neurosci, 1999
- Developmental expression of  **synaptophysin** ,  **synapsin I**  and syntaxin in the rat retina.  Ref: 9352109 Brain Res Dev Brain Res, 1997
- **Synapsin I**  was purified by conventional means from human and porcine brain and  **synaptophysin**  was purified by immunoaffinity chromatography from porcine brain.  Ref: 9692851 J Immunol Methods, 1998
- In order to investigate the effects of antidepressant compounds on SVP mRNA levels, the expressions of  **synaptophysin** , synaptotagmin, VAMP, and  **synapsin I**  were analysed by in situ hybridization in rats which had been treated with desipramine, fluoxetine, tranylcypromine, or saline.  Ref: 14708000 Pharmacogenomics JPharmacogenomics J, 1998
- the levels of  **synaptophysin**  p38,  **synapsin I**  and sv2... correlate with the extent of FM1 43 labelling at synapses.  Ref: 9153578 Eur J Neurosci, 1997
- SV2,  **synapsin I** , and  **synaptophysin**  revealed the organization of the barrel field in the neonate.  Ref: 8915833 J Comp Neurol, 1996
- BDNF also increased the levels of the vesicle proteins  **synaptophysin** , synaptobrevin, and synaptotagmin, without affecting the presynaptic membrane proteins syntaxin and SNAP 25, or the vesicle binding protein  **synapsin I** .  Ref: 11483592 J Biol Chem, 2001
- **Synaptophysin**  and  **synapsin I** , another synaptic vesicle protein, are also expressed by retinoic acid induced neuronally differentiated P19 teratocarcinoma cells.  Ref: 12492469 Eur J Biochem, 2003
- Parallel increases in  **synaptophysin**  immunoreactivity were not observed, thus suggesting a selective induction of  **synapsin I**  after ischemia.  Ref: 10196445 Brain Res, 1999
- Using the ELISA, neither  **synapsin I**  nor  **synaptophysin**  could be determined in serum or cerebrospinal fluid CSF from healthy donors or patients suffering various neurological disorders or pheochromocytomas.  Ref: 9692851 J Immunol Methods, 1998
